# Papillary thyroid carcinoma measuring 1.0 cm or less: an epidemiological and clinicopathological study

**DOI:** 10.3389/fonc.2025.1678065

**Published:** 2025-11-06

**Authors:** Imad A. El Hag, Raghad Tallab

**Affiliations:** Department of Pathology and Laboratory medicine, Security Forces Hospital, Riyadh, Saudi Arabia

**Keywords:** PTMC, incidental, non-incidental, high-risk, subtypes

## Abstract

**Background:**

The incidence of papillary thyroid microcarcinoma (PTMC) measuring 1 cm or less has noticeably increased over the past 30 years, particularly among clinically significant subgroups that are diagnosed chiefly preoperatively.

**Methods:**

This retrospective study investigated the rate of occurrence and the pathological high-risk features of PTMC discovered incidentally during thyroidectomies for benign thyroid diseases, as well as those diagnosed preoperatively (non-incidental), in a sample of 1,408 thyroidectomies.

**Results:**

PTMC accounted for 30.5% of all resected malignant thyroid tumors, with 53% being incidental and 47% non-incidental. The incidence of incidental PTMC has increased twofold over the past seven years, from 4.5% to 9.0%, while the incidence of the non-incidental subgroup has increased tenfold, from 0.9% to 9.0%. Compared to incidental cases, non-incidental cases were more likely to affect males, exhibited significantly larger tumors (7.2 mm versus 3.7 mm), and had higher rates of multiplicity (49.4% vs. 23.6%) and bilaterality (36.7% vs. 16.7%). Non-incidental cases also had higher rates of lymph node metastasis (25.9% vs. 4.2%) and greater margin involvement (34.4% vs. 16.7%). Extrathyroidal extension occurred exclusively in approximately 2.5% of the non-incidental cases. The most aggressive PTMC subtype is the tall cell subtype (TCS), followed by the classic subtype, which is observed in 30% and 29% of non-incidental cases, respectively. Follicular subtypes exhibit indolent behavior, as observed in 11% of non-incidental cases. An account of the TCS morphology is also provided.

**Conclusion:**

The incidence of PTMC has dramatically increased in the non-screened population. Male sex, tumor size >5 mm, and TCS subtype are risk factors for aggressive behavior.

## Introduction

Papillary thyroid carcinoma (PTC) equal to or less than 1.0 cm, hereafter referred to as papillary thyroid microcarcinoma (PTMC), was previously viewed as a subtype of PTC. However, the 2022 WHO classification of thyroid neoplasms no longer recognizes PTMC as a distinct subtype (1, 2). The past terminologies used to describe these tumors *include incidental carcinoma, latent carcinoma, occult carcinoma, papillary microcarcinoma, and papillary microtumor*, reflecting the ongoing controversy regarding their biological behavior. PTMC has long been considered an indolent subtype of PTC with an excellent prognosis (3). A significant increase in these tumors has been observed worldwide over the last 30 years, likely attributable to advances in imaging techniques and the routine use of ultrasound (US) and fine-needle aspiration (FNA) in the surveillance of patients with thyroid nodules (4). Amidst this epidemic, a subset of PTMC tumors has emerged as clinically significant, characterized by aggressive pathological features, leading to local recurrence, distant metastasis, and progression to high-grade carcinoma with adverse outcomes (2). As a result, the 2022 WHO now categorizes PTMCs as pT1 carcinomas, positioning them as PTCs greater than 1 cm in terms of diagnostic work-up, risk stratification, and management guidelines.

The natural history of PTMC shows that these tumors are quite different. The overall prevalence of PTMC in autopsy studies was 11.5%. In contrast, it is 0.39% in mass screening of healthy individuals of both sexes, 3.5% in women undergoing breast screening, and 5.0% in patients who have undergone benign thyroidectomy procedures (5–7). Each of these diverse prevalence rates is over a thousand-fold greater than the worldwide incidence of thyroid cancer, which is 14.6 per 100,000 annually (8). This suggests that most PTMCs are latent tumors that grow slowly and silently, often remaining hidden in the thyroid glands of patients who die with them, not because of them. Only a small proportion of PTMCs were clinically evident. Compared to incidental or latent tumors, clinically overt or non-incidental tumors are generally larger (>5 mm), exhibit higher BRAF mutation rates, have an increased risk of lymph node metastasis and local recurrence, and can even lead to death (9–13). It is not only the size, but also the genetics, morphological subtype, and clinical characteristics that play an essential role (12, 14, 15).

These tumors have long been regarded as nonaggressive. With the increase in their diagnosis, debate over their management has arisen, and opinions are divided into active surveillance, deferred, or immediate surgical intervention, considering their costs, psychological impacts, associated morbidities, and mortalities (16–18).

This study describes the epidemiological, pathological, and clinical characteristics of PTMCs, as well as the trend in their diagnosis at our institution over a seven-year period. Our goal was to enhance our understanding of PTMC behavior and inform the development of appropriate management strategies.

## Materials and methods

PTMC cases diagnosed during complete or partial thyroidectomies performed between 2018 and the first quarter of 2025 were retrieved from the archives of the Department of Laboratory Medicine and Pathology at the Security Forces Hospital in Riyadh, Kingdom of Saudi Arabia. Based on the indications for surgery, the cases were categorized into incidental or non-incidental subgroups.

Cases identified incidentally by the pathologist during the histopathological examination of thyroids removed because of benign conditions, such as Graves’ disease, nodular goiter, autoimmune thyroiditis, adenomas, follicular tumors, or parathyroid disease, with no preoperative suspicion of malignancy, were deemed incidental. In contrast, non-incidental cases were preoperatively diagnosed as malignant based on highly suspicious ultrasound findings consistent with a TIRADS score of 5 or a suspicious or positive FNA diagnosis (Bethesda categories IV, V, and VI).

**Figure 1:**
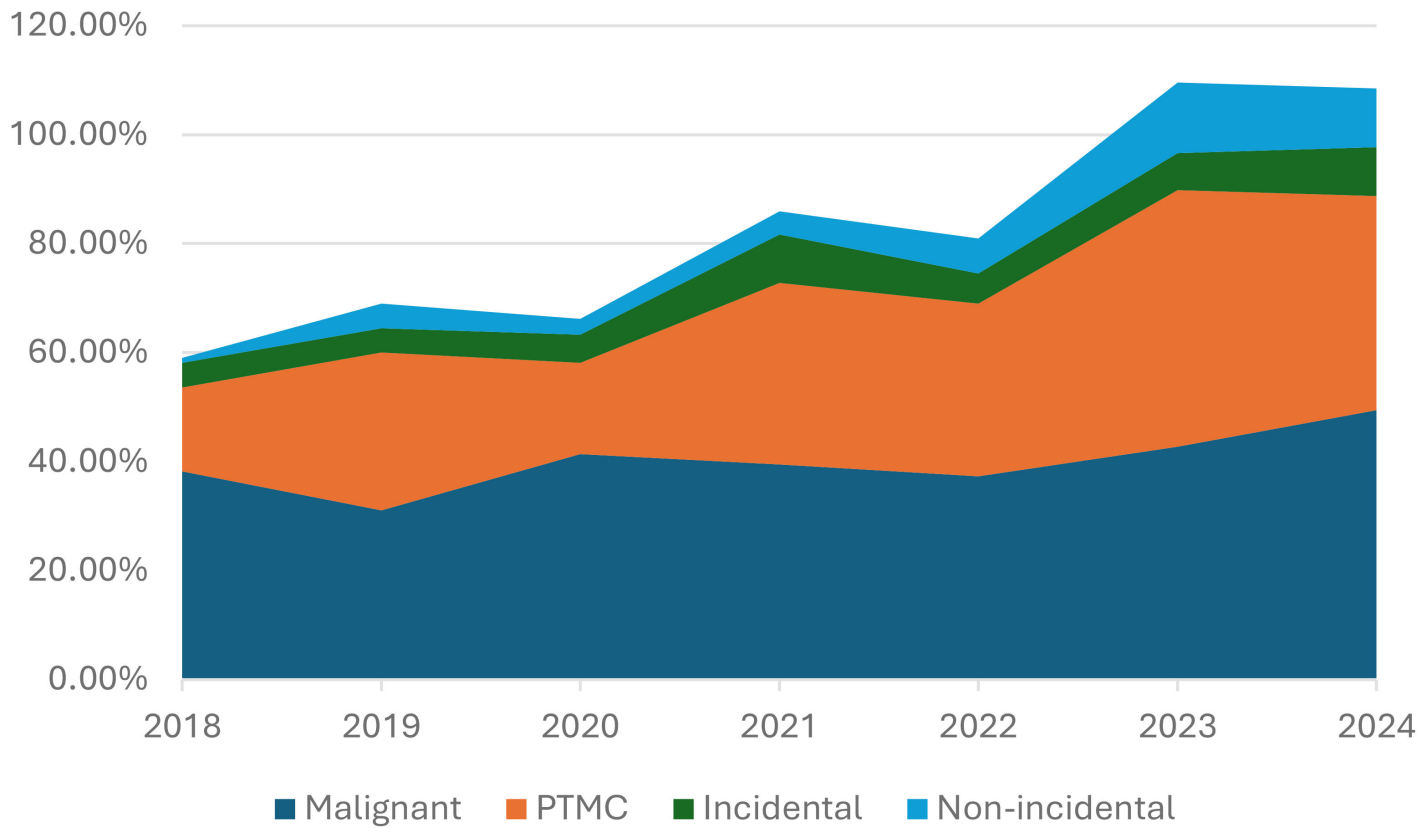
Incidence of malignant thyroid tumors, PTMC, and its two subgroups, incidental and non-incidental, over the years.

The trend in PTMC diagnosis and its incidental and non-incidental subgroups was assessed over the years as a proportion of the total number of malignant cases and compared accordingly (**Figure 1**).

Sociodemographic data, including sex and age at diagnosis were recorded. The tumor size (mm), multiplicity, laterality, morphological subtypes, extrathyroidal extension, and lymph node status were determined. Only tumors measuring ≤ 10 mm in diameter were included in this study. A PTMC was classified as multifocal when it presented with more than one focus or bilateral involvement.

The inked margin status was documented as positive or negative. A margin was classified as positive if the tumor reached the inked margin, and a cancer with the smallest possible free margin was classified as negative. The number of lymph nodes examined and whether central or lateral was recorded, along with their metastatic status and the ratio of positive lymph nodes to the total number of examined lymph nodes (PLR). Extrathyroidal extension was considered only when the operating surgeon noted adherence of the tumor to the strap muscles, as confirmed by subsequent histopathological examination.

The slides were reviewed by two pathologists, and all PTMC cases were subtyped based on predetermined morphological features, as illustrated in the latest WHO classification of thyroid tumors (**Table 1**). The frequencies of various morphological subtypes were determined from the total number of tumors identified (**Table 2**). The relationship between PTMC morphological subtypes and subgroups and pathological features associated with a high risk of aggressive behavior, such as lymph node metastasis, margin involvement, extrathyroidal extension, tumor multiplicity, and bilaterality, was assessed and compared using the dominant tumor in each case (**Tables 3**, **4**). A dominant tumor was defined as the largest multifocal tumor.

**Table 1:** Diagnostic criteria for each papillary thyroid carcinoma subtype.

Subtype	Diagnostic features
Classic	Papillae with broad fibro-vascular core in any proportion with diffuse papillary nuclear features.
Follicular	Exclusively follicular growth pattern with papillary nuclear features.
Tall cell	≥ 30% tall cells – eosinophilic cells, height 2–3 times width, distinct cytoplasmic border and tram tracking arrangement.
Oncocytic	Exclusively oncocytic, papillary, solid or follicular.
Warthin-like	Papillary oncocytic in a lymphoplasmacytic background
Solid	> 30% solid or trabecular pattern.

**Table 2:** The table shows the morphological type of PTMC and its relative proportion in descending order.

Morphological type	Percent
Classic	82 (32.0%)
Tall	69 (27.0%)
Follicular	68 (26.6%)
Oncocytic	18 (07.0%)
Solid	11 (04.3%)
Warthin-like	08 (03.1%)
Total	256

**Table 3:** The frequency of PTMC subtypes in incidental and non-incidental groups.

Type	All tumors		Incidental			Non-incidental		
	No.	%	Type	No.	%	Type	No.	%
Classical	57	33.9	Classic	28	30.4	Tall cell	30	38.0
Tall	46	27.4	Follicular	29	32.6	Classic	29	36.7
Follicular	40	23.9	Tall cell	16	18.0	Follicular	11	13.8
Oncocytic	12	7.0	Oncocytic	8	9.0	Oncocytic	4	5.1
Solid	9	5.4	Solid	5	5.6	Solid	4	5.1
Warthin-like	4	2.4	Warthin-like	3	3.4	Warthin-like	1	1.3
Total	168			89			79	

**Table 4:** The clinical and pathological high-risk features.

Parameters	Type	Type	P values
	Incidental N = 89	Non-incidental N = 79	
Age	44.9 ± 11.3	44.3±11.9	= .863
% Male	14.5%	15.2%	<.001
Tumor size in mm	3.7 ± 1.8	7.2 ± 2.0	<.001
Multiplicity	21(23.6%)	39 (49.4%)	<.001
Bilaterality	15 (16.7%)	29 (36.7%)	<.001
Positive margins	15 (16.7%)	27 (34.2%)	<.001
Extrathyroidal extension	0 (0%)	2 (2.5%)	=.500
Lymph node metastasis	2/48 (4.2%)	14/54 (25.9%)	<.001

Comparison between incidental and non-incidental subgroups.

A morphological and epidemiological account of the tall cell subtype (TCS) was provided. The risk factors for aggression, size, lymph node status, and margin status at diagnosis were compared with those of the more common subtypes, namely the classic and follicular subtypes (**Table 5**).

**Table 5:** Comparison of rates of high-risk features between different PTMC subtypes.

Subtypes	Classic	Follicular	Tall cell	P values
Age years	42±14	44±10	44±9	=.26
Size in mm	5.7±2.4	3.9±2.3	6.0±2.4	<.001
Encapsulation	14/57 (24.5%)	6/40 (15%)	4/46 (8.7%)	=.17
Margin	12/57(21.0%)	7/40 (1.1%)	21/46 (46.6)	=.011
LN metastasis	4/32 (11.4%)	4/20 (16.6%)	6/30 (20%)	=.352

The sociodemographic and pathological parameters mentioned above were analyzed using the SPSS software. Two-tailed χ² or Fisher’s exact tests were used to compare proportions, while the two-tailed t-test was applied to compare means, such as age, tumor size, and lymph node counts, as indicated in the results.

## Results

This study included 168 consecutive cases of PTMC diagnosed between 2018 and the first quarter of 2025, identified from a total of 1,408 partial or total thyroidectomies. The patients ages ranged from 22 to 89 years, with a mean age of 44 years. The cohort consisted of 143 females and 25 males, with a female-to-male ratio of approximately 6:1.

Eighty-nine cases (53%) were classified as incidental and identified during histopathological examination of thyroids removed for benign conditions, with no preoperative suspicion of malignancy. The remaining 79 cases (47%) were categorized as non-incidental, all of which had undergone radiological, cytological, or both types of preoperative testing that were suspicious for malignancy.

Of the 1,408 thyroidectomies performed, 554 were malignant (39.3%) and the remaining 854 were benign (59.7%). The prevalence of PTMC among malignant cases was 30.5%. The prevalence rates of incidental and non-incidental PTMC were 16.2% and 14.3%, respectively.

**Figure 1** illustrates the incidence trends for PTMC and its subgroups. The overall percentage of PTMC, as well as that of the incidental subgroup among malignant tumors, increased steadily over the study period by a factor of three and two, respectively. In contrast, non-incidental PTMC showed a ten-fold increase, rising from 0.9% to 10.8% over the same period.

**Figure 2:**
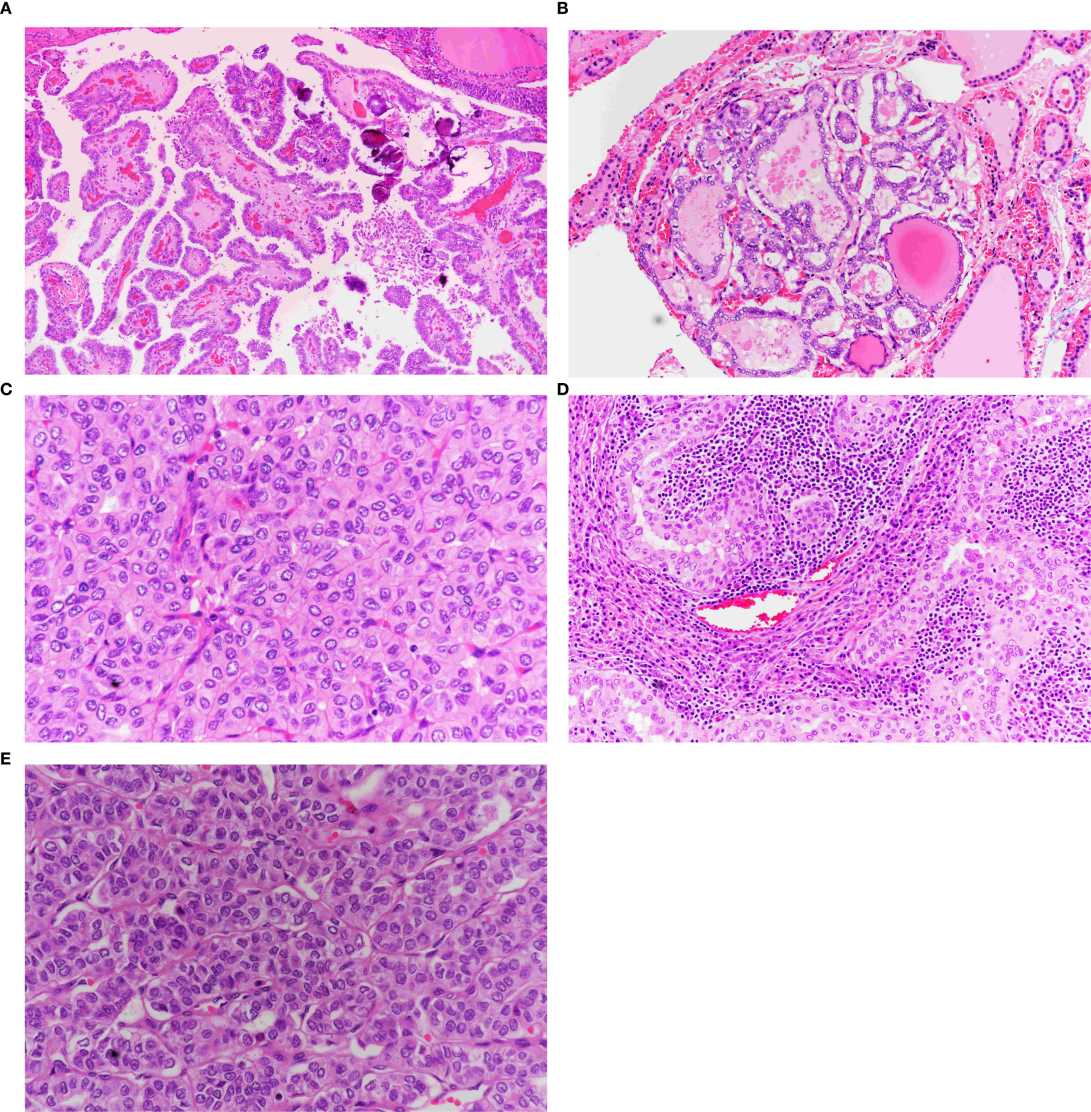
Morphological subtypes of PTMC. **(A)** Classic papillary thyroid microcarcinoma. Note the papillae with broad fibrovascular cores covered by follicular cells exhibiting diffuse papillary nuclear features (×40). **(B)** Follicular variant of PTMC. Exclusively follicular growth pattern with characteristic papillary nuclear features (×40). **(C)** Oncocytic PTMC. Solid and trabecular growth pattern composed entirely of oncocytic cells with papillary nuclear features (×40). **(D)** Warthin-like PTMC. Papillary oncocytic growth within a lymphoplasmacytic background (×40). **(E)** Solid variant of PTMC. Solid, follicular, and trabecular growth patterns with papillary nuclear features (×40).

A total of 256 PTMC tumor foci were identified in 168 cases. The morphological subtypes and frequencies are presented in **Table 2**. Regardless of the subgroup, the classical subtype was the most common (32%), followed by the tall cell (27.0%) and follicular (26.6%) subtypes. These three subtypes account for 85.6% of all PTMC tumors. The minor subtypes included oncocytic, solid, and Warthin-like subtypes (**Figure 2**). The diffuse sclerosing and columnar subtypes were not observed in this study.

When cases were categorized into incidental and non-incidental subgroups and only the dominant tumor (the largest focus in multifocal cases) was considered (**Table 3**), a notable difference in the distribution and frequency of morphological subtypes was observed between the two groups (p = .014). The tall cell subtype was most common in the non-incidental subgroup, followed by the classical subtype. Conversely, the classical subtype was the most prevalent in the incidental subgroup, followed by the follicular subtype.

**Table 4** compares the demographic and pathological features of incidental and non-incidental PTMC using univariate analysis (χ²test or t-test, as appropriate). There was no statistically significant difference in age between the two groups. However, more males were reported in the non-incidental group. Although the difference was small, it was statistically significant (p = <.001). Compared to incidental PTMC, non-incidental PTMC cases had larger tumors (mean size 7.2 mm vs. 3.7 mm), were more often bilateral (36.7% vs. 16.7%), and more frequently multifocal (49.4% vs. 23.3%). Non-incidental PTMC also showed a higher tendency for regional lymph node metastasis, with a rate of 25.9%, six times higher than the 4.2% seen in the incidental group. The majority of affected lymph nodes are of the central type, 14 out of 16 (88%), with only 12% being lateral. Margin involvement occurred in 34.2% of non-incidental cases compared to 16.7% in incidental cases. Although uncommon, extrathyroidal extension was found only in the non-incidental subgroup (2.5%).

Cervical lymph nodes were identified and removed in 102 patients: 48 of 89 incidental PTMC cases (54%) and 54 of 79 non-incidental PTMC cases (68%). The average number of lymph nodes harvested was four (range: 1–13) in the incidental group and six (range: 1–48) in the non-incidental group. These differences were statistically significant, with a p-value less than 0.05. Lymph node metastases were found in 16 cases (9.5%): 2 in the incidental subgroup and 14 in the non-incidental subgroup. The lymph node ratio (PLR), defined as the number of positive lymph nodes divided by the total number of examined lymph nodes, was less than 0.3 in 4 cases (25%) and greater than 0.3 in 12 cases (75%), regardless of the subgroup.

**Figure 3:**
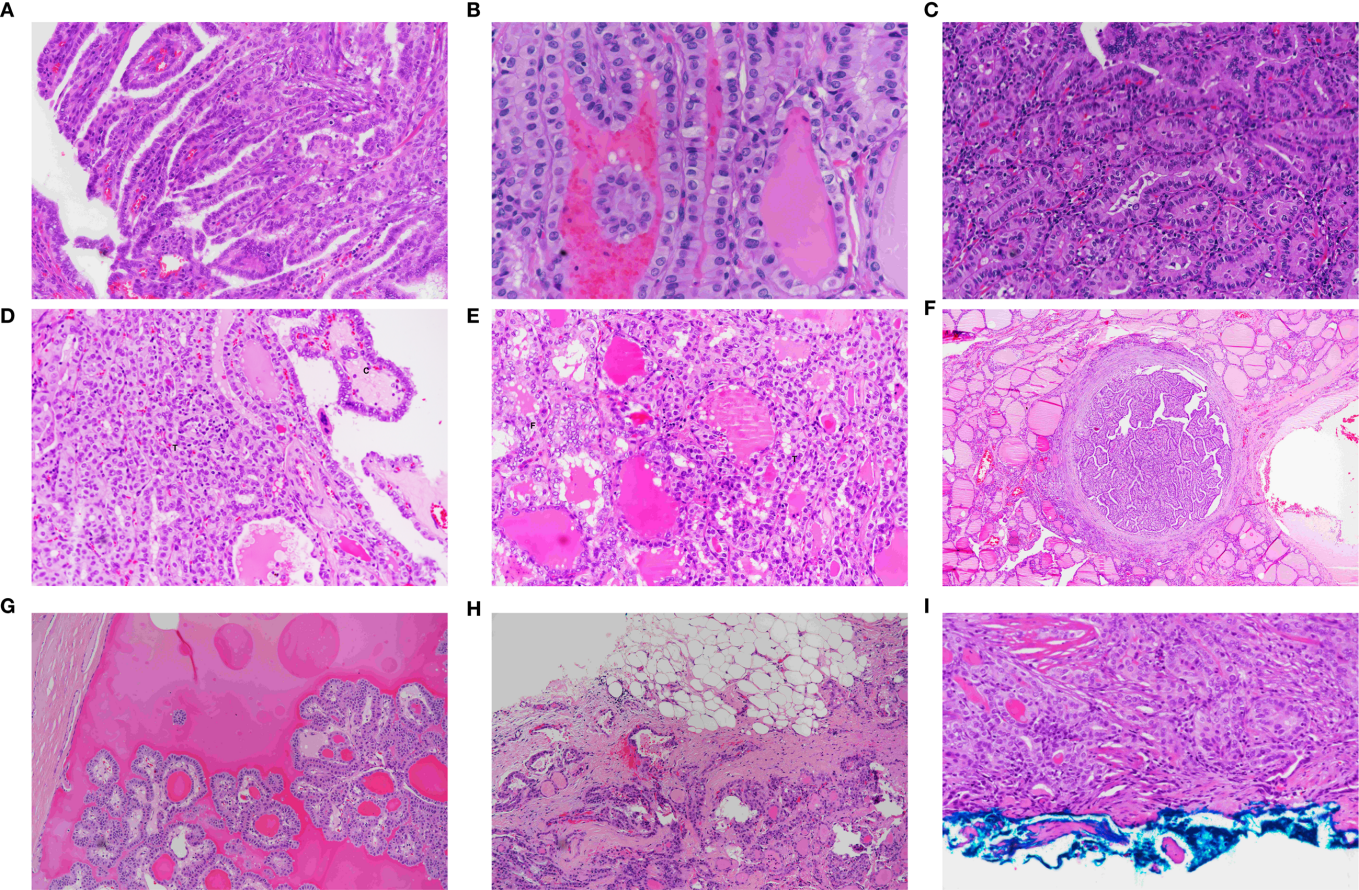
Morphological features of papillary thyroid carcinoma, tall cell subtype. **(A)** TCS showing tall, slender papillae arranged in parallel, creating a tram-track appearance (×40). **(B)** Tram-track appearance in TCS (×40). **(C)** TCS composed of tall eosinophilic cells with height 2–3 times their width, distinct cytoplasmic borders, and basal, non-stratified nuclei (×40). **(D)** Mixed TCS (T) and classic (C) PTMC (×40). **(E)** Mixed follicular (F) and TCS (T) papillary microcarcinoma (×40). **(F)** Fully encapsulated TCS showing tram-track appearance at low power (×20). **(G)** Cystic TCS with papillary tumor fragments floating in a cystic space (×40). **(H)** Infiltrative growth pattern characteristic of TCS (×20). **(I)** TCS involving the inked surgical margin (×40).

Hematoxylin and eosin sections from 46 patients with a dominant tumor diagnosed as the tall cell subtype (TCS) were available for review. These included 40 females and six males, with a female-to-male ratio of 6:1. The mean age of the patients was 47 years (range, 25–60 years). Sixteen cases (34.8%) were discovered incidentally, while 30 (65.2%) were diagnosed as malignant before surgery. Non-incidental TCS tumors were significantly larger than incidental ones (7.2 ± 1.9 mm vs. 3.8 ± 1.4 mm; p <.001). These tumors were rarely encapsulated or cystic, with complete encapsulation and cystic changes observed in only 8.7% and 2.2% of the cases, respectively (**Figures 3F, G**).

TCS primarily showed infiltrative growth regardless of tumor size, with a 46.6% rate of reaching the inked margin. (**Figures 3H, D**). Extrathyroidal extension was seen in one case. These tumors were often multifocal (45.7%) and bilateral (28.3%) and were linked to regional lymph node metastasis (20%).

Compared to the classical and follicular subtypes, TCS was associated with a larger tumor size, more frequent lymph node metastasis, and higher rates of margin involvement (**Table 5**).

Histologically, the TCS consisted of slender, longitudinally oriented papillary structures with narrow fibrovascular cores (**Figure 3A**). Depending on the sectioning plane, adjacent papillae demonstrated tram-track, trabecular, tubular, or mixed appearance (**Figures 3B, C**). The tumor cells were taller than wide, with eosinophilic cytoplasm and distinct cell borders (**Figure 3C**). The* nuclei were typically located basally with occasional pseudostratification. All tumors demonstrated the classic nuclear features of papillary thyroid carcinoma, including nuclear enlargement, grooves, chromatin clearing, and intranuclear inclusions. Psammoma bodies were observed in 50% of tumors.

The tumors were composed of purely tall cells in 42 cases (91%) and were mixed with classical (**Figure 3D**) or follicular (**Figure 3E**) patterns in 4 cases (9%). In mixed tumors, the non–tall cell component varies between 10% and 50% of the tumor size.

## Discussion

Thyroid cancer (TC) is the most common endocrine malignancy. As of 2022, it is ranked seventh in global cancer incidence and 24th in cancer-related mortality, with an annual incidence of 10.1 per 100,000 in females and 3.1 per 100,000 in males. The mortality rate is 0.5/100,000 individuals (19). This striking contrast between the incidence and mortality highlights the indolent nature of the disease. Thyroid cancer is more prevalent in countries with a high Human Development Index (HDI) than in those with a low HDI. Globally, its incidence is projected to increase by 29.9% by 2040, while mortality is expected to increase by 64% during the same period, primarily in low-HDI countries (20).

Thyroid cancer is the third most common cancer in Saudi Arabia. Its incidence has steadily increased over the past 30 years in both sexes from 2 to 14 per 100,000 annually. Mortality has also risen nearly threefold, with both incidence and mortality increasing more prominently in males than in females (21).

More than 50% of newly diagnosed thyroid cancers are tumors measuring ≤1 cm (22). The rapid increase in PTMC is primarily attributed to the widespread use of advanced imaging and ultrasound-guided FNA in patients with thyroid nodules. However, environmental risk factors, including radiation exposure, iodine deficiency, and lifestyle choice, may also contribute to this trend.

Incidental PTMC has been detected in autopsy studies, mass screenings, benign thyroidectomies, and surveillance studies, providing insights into its natural history. Autopsy studies reported a PTMC detection rate ranging from 2.9% in Chile to 35.6% in Finland, with an average of 11.5% (5). These tumors are typically smaller than 5 mm, discovered in the seventh decade of life, and affect both males and females equally. In our study, the detection rate of PTMC in benign thyroidectomies was 10.4%, consistent with the reported ranges of 2.3%–14.1% (7, 23). Similarly, these tumors are usually <5 mm, more frequent in females, and most often discovered in the fifth decade of life.

The detection rates of PTMC during breast cancer screening in Korea, the United States, and Japan, as well as in healthy individuals in Japan, range from 0.39% to 3.5% (4, 24, 25). In these settings, tumors are typically >5 mm, occur more frequently in women, and are diagnosed predominantly in the fifth decade of life. The apparent female predominance in mass screening and benign thyroidectomy specimens, in contrast to the equal sex distribution in autopsy studies, may reflect a selection bias. There are two possible explanations for this discrepancy: either tumors regress more frequently in aging females or, more likely, a biologically driven event sustains and promotes progression in younger females. Evidence from active surveillance studies in Japan supports this hypothesis (25). In most cases, PTMC remains stable or regresses, with only a minority demonstrating disease progression. Tumor growth ≥3 mm occurred in 6.4% and 15.6% of cases at five and ten years, respectively. After ten years of follow-up, tumor enlargement was observed in 8% of the cases, and new lymph node metastases developed in 3.8%. Progression was more common in young to middle-aged women, whereas tumors in men tended to regress over time.

As mentioned above, it becomes evident that PTMC is a common and primarily indolent tumor, typically small, often <5 mm in size, with an equal sex distribution; however, only a small subset progresses, primarily in younger women, and becomes clinically significant. The role of estrogen in the maintenance and progression of these tumors in women has been discussed previously (26).

In our study, the incidence of incidental PTMC has steadily increased over the past seven years, doubling from 4.5% in 2018 to 9.0% in 2023. Meanwhile, the increase in non-incidental PTMC was significant, tenfold, from 0.9% to 10.8% during the same period. This surge in incidental tumors likely reflects the overall increase in thyroid cancer cases in Saudi Arabia and other regions. However, the notable increase in non-incidental PTMC raises an important question: Is this rise in a non-screened population solely due to the more frequent use of ultrasound and FNA, or are other biological or environmental factors contributing?

Although some may interpret this rise as overdiagnosis leading to higher healthcare costs and postoperative morbidities, it can also be viewed positively, as early detection, with all its benefits, has become possible owing to advancements in healthcare and diagnostic technology.

We reported an average tumor size of 7.2 mm in the non-incidental subgroup, which is comparable to the 6.9 mm average reported in a meta-analysis study (9). Both figures align with findings from a surveillance study, which observed that tumors likely to progress were initially approximately 7 mm in size (4). Considering the commonly reported tumor size of roughly 7 mm in the non-incidental subgroup, with a standard deviation of ±2.0 mm (**Table 2**), the smallest significant size was 5 mm. This threshold may represent a critical point for progression and could serve as a cutoff for clinical staging and quality assurance in preoperative diagnostic testing.

The tall cell subtype (TCS) is the second most aggressive PTC variant after the diffuse sclerosing subtype (27, 28). Aggressive variants, including TCS, exhibit high rates of recurrence, lymph node metastasis, and extrathyroidal extension and may even show more aggressive clinical behavior than tumors >1 cm (28). Although diffuse sclerosing is the most reported aggressive subtype in other studies, our series identified TCS as the predominant aggressive variant. None of our patients had the diffuse sclerosing subtype. These findings are consistent with those of the pioneering study by Gubbiotti et al. (14).

In our series, TCS showed the highest rates of lymph node metastasis (20%) and margin involvement (46.6%), along with high tumor volumes and infiltrative growth. The classic, oncocytic, and solid subtypes follow TCS, whereas the follicular subtype appeared to be the least aggressive.

The clinicopathological features of TCS have been primarily studied in tumors > 1 cm (14). TCS is known to behave more aggressively with higher recurrence rates and poorer survival outcomes. This remains true regardless of the proportion of tall cells required for diagnosis, which varies between 10% and 75%, with 30% being the most commonly used proportion. Cohort studies on PTMC have not consistently reported the proportion of tall cells in their cases; however, a 30% cut-off has been recommended for diagnosis (14, 27, 28).

In our series, 91% of TCS cases consisted entirely of tall cells, whereas 9% had mixed components (follicular or classic), comprising up to 50% of the tumor. The presence of these additional patterns is considered a form of differentiation typically observed in larger tumors and is not expected to impact tumor behavior significantly. Although most of our TCS cases involved purely tall cells, we agree that a proportion as low as 10% is clinically relevant and should be reported.

If we consider two major risk factors, lymph node metastasis and tumor multiplicity (12), our findings in patients with TCS ≤1 cm (with rates of 20% and 46.6%, respectively) fall within the reported range for tumors >1 cm (23.3–83.3% and 47%, respectively), supporting the notion that their behavior is comparable (29). An increase in TCS incidence has been observed globally in recent decades (29), and TCS ≤1 cm constitutes a substantial proportion of PTMC cases in the literature (14, 27, 28). This may reflect a growing awareness and recognition, although an actual biological increase cannot be ruled out. At the molecular level, TCS is characterized by a higher prevalence of *BRAF* and *TERT* mutations, *RET*/*PTC3* rearrangements, and overexpression of *c-MET, p53*, and *MUC1* than classical subtypes (4, 29). These molecular alterations are likely to contribute to their aggressive behavior.

We suspect that TCS mainly arises peripherally in the thyroid and exhibits infiltrative growth, which is why it reaches the margins more frequently than other subtypes, especially follicular cells. The latter was least likely to involve the margin (**Table 5**). This suggests a central origin within the goitrous nodule for the follicular subtype.

At our institution, all PTC cases were surgically managed, regardless of size, with no role of active surveillance. In 2023, more than 47% of thyroid cancer surgeries were performed for tumors ≤1 cm. The sharp rise in non-incidental PTMC, indicating an increased disease burden, may represent the first such trend to be reported in the region. Active surveillance with or without deferred surgery has been advocated as an alternative to immediate surgical intervention. It has shown comparable effectiveness to surgery while offering the benefits of lower cost and reduced morbidity (30). The disease tends to progress in women younger than 40 years and regress in older males, making the latter good candidates for observation and the former more suitable for deferred surgery. This robust evidence supporting active surveillance comes primarily from Japanese studies. While Western experience remains preliminary, there are no contraindications for adopting this approach (31).

Despite the overall indolent behavior of PTMC reported over the past few decades (29), tumors with TCS ≤1 cm constitute a significant proportion of PTMC in various published studies (4, 27, 28). PTMC has a disease-related mortality of 1.0%, lymph node recurrence of 5.0%, and a distant metastasis rate of 2.5%, suggesting a behavior similar to tumors >1 cm (12). These findings suggest that active surveillance may not be the most effective approach, particularly in a non-screened population, such as ours. Similarly, in the present study cohort, lymph node metastasis was observed in 9.5% of PTMC cases, with 75% of these cases showing a lymph node ratio (PLR) greater than 0.3. This places our population within a recurrence risk range similar to that of PTCs larger than 1.0 cm (32, 33). This study has several limitations that should be acknowledged, including its retrospective nature, small sample size from a single institution, absence of molecular testing, and incomplete clinical follow-up data, which should be considered when interpreting the results.

## Conclusion

The incidence of non-incidental PTMC has been steadily increasing over the years in non-screened populations. These tumors are mostly larger than 5 mm in size, and more males are affected than in the incidental subgroup, often exhibiting more aggressive behavior. TCS is the most frequent aggressive subtype, behaving similarly to tumors >1 cm in terms of clinical course and outcome, and should be managed accordingly.

## Data Availability

The raw data supporting the conclusions of this article will be made available by the authors, without undue reservation.

## References

[1] Kakudo K Bychkov A Bai Y Li Y Liu Z Jung CK . The new 4th edition World Health Organization classification for thyroid tumors: Asian perspectives. Pathol Int. (2018) 68:641–64. doi: 10.1111/pin.12737, PMID: 30537125

[2] Baloch ZW Asa SL Barletta JA Ghossein RA Juhlin CC Jung CK . Overview of the 2022 WHO classification of thyroid neoplasms. Endocr Pathol. (2022) 33:27–63. doi: 10.1007/s12022-022-09707-3, PMID: 35288841

[3] Baloch ZW LiVolsi VA . Microcarcinoma of the thyroid. Adv Anat Pathol. (2006) 13:69–75. doi: 10.1097/01.pap.0000213006.10362.17, PMID: 16670460

[4] Xu S Han Y . The overdiagnosis of thyroid micropapillary carcinoma: the rising incidence, inert biological behavior, and countermeasures. J Oncol. (2021) 2021:5544232. doi: 10.1155/2021/5544232, PMID: 34306078 PMC8285179

[5] Lee YS Lim H Chang HS Park CS . Papillary thyroid microcarcinomas are different from latent papillary thyroid carcinomas at autopsy. J Korean Med Sci. (2014) 29:676–9. doi: 10.3346/jkms.2014.29.5.676, PMID: 24851024 PMC4024958

[6] Ito Y Miyauchi A Oda H . Low-risk papillary microcarcinoma of the thyroid: a review of active surveillance trials. Eur J Surg Oncol. (2018) 44:307–15. doi: 10.1016/j.ejso.2017.03.004, PMID: 28343733

[7] Peluso G Masone S Campanile S Criscitiello C . Incidental papillary microcarcinoma on 1777 surgically treated patients for benign thyroid disease: a noninstitutional experience and literature review. Memo. (2020) 13:126–33. doi: 10.1007/s12254-019-00567-y

[8] Bray F Laversanne M Sung H Ferlay J Siegel RL Soerjomataram I . Global cancer statistics 2022: GLOBOCAN estimates of incidence and mortality worldwide for 36 cancers in 185 countries. CA Cancer J Clin. (2024) 74:229–63. doi: 10.3322/caac.21834, PMID: 38572751

[9] Mehanna H Al-Maqbili T Carter B Martin E Campain N Watkinson J . Differences in the recurrence and mortality outcomes rates of incidental and nonincidental papillary thyroid microcarcinoma: a systematic review and meta-analysis of 21,329 person-years of follow-up. J Clin Endocrinol Metab. (2014) 99:2834–43. doi: 10.1210/jc.2013-2118, PMID: 24828487

[10] Song YS Kang BH Lee S Yoo SK Choi YS Park J . Genomic and transcriptomic characteristics according to size of papillary thyroid microcarcinoma. Cancers (Basel). (2020) 12:1345. doi: 10.3390/cancers12051345, PMID: 32466217 PMC7281223

[11] Ghossein R Ganly I Biagini A Robenshtok E Rivera M Tuttle RM . Prognostic factors in papillary microcarcinoma with emphasis on histologic subtyping: a clinicopathologic study of 148 cases. Thyroid. (2014) 24:245–53. doi: 10.1089/thy.2012.0645, PMID: 23745671

[12] Chow SM Law SC Chan JK Au SK Yau S Lau WH . Papillary microcarcinoma of the thyroid: prognostic significance of lymph node metastasis and multifocality. Cancer. (2003) 98:31–40. doi: 10.1002/cncr.11442, PMID: 12833452

[13] Piana S Ragazzi M Tallini G de Biase D Ciarrocchi A Frasoldati A . Papillary thyroid microcarcinoma with fatal outcome: evidence of tumor progression in lymph node metastases—report of 3 cases, with morphological and molecular analysis. Hum Pathol. (2013) 44:556–65. doi: 10.1016/j.humpath.2012.06.019, PMID: 23079204

[14] Gubbiotti MA Livolsi V Montone K Baloch Z . Papillary thyroid microcarcinomas: does subtyping predict aggressive clinical behavior? Hum Pathol. (2021) 114:28–35. doi: 10.1016/j.humpath.2021.04.015, PMID: 33971214

[15] Osborne D Choudhary R Vyas A Kampa P Abbas LF Chigurupati HD . Hashimoto’s thyroiditis effects on papillary thyroid carcinoma outcomes: a systematic review. Cureus. (2022) 14:e28054. doi: 10.7759/cureus.28054, PMID: 36120263 PMC9476374

[16] Shaha AR Tuttle RM . Active surveillance for micropapillary thyroid carcinoma: a clinical review. Gland Surg. (2024) 13:100–7. doi: 10.21037/gs-22-558, PMID: 38323232 PMC10839699

[17] Issa PP Munshi R Albuck AL Omar M Abu Alhuda RF Metz T . Recommend with caution: a meta-analysis investigating papillary thyroid carcinoma tumor progression under active surveillance. Am J Otolaryngol. (2023) 44:103994. doi: 10.1016/j.amjoto.2023.103994, PMID: 37607459

[18] Aryanti C Sudarsa IW Adiputra P . Meta-analysis of the outcomes in active surveillance and surgical approach for micropapillary thyroid carcinoma. Asian Pac Environ Cancer. (2021) 4:25–31. doi: 10.31557/apjec.2021.4.1.25-31

[19] Pizzato M Li M Vignat J Laversanne M Singh D La Vecchia C . The epidemiological landscape of thyroid cancer worldwide: GLOBOCAN estimates for incidence and mortality rates in 2020. Lancet Diabetes Endocrinol. (2022) 10:264–72. doi: 10.1016/S2213-8587(22)00035-3, PMID: 35271818

[20] Shank JB Are C Wenos CD . Thyroid cancer: global burden and trends. Indian J Surg Oncol. (2022) 13:40–5. doi: 10.1007/s13193-021-01429-y, PMID: 35462648 PMC8986939

[21] Flemban AF Kabrah S Alahmadi H Alqurashi RK Turaes AS Almaghrabi R . Patterns of thyroid cancer mortality and incidence in Saudi Arabia: a 30-year study. Diagnostics (Basel). (2022) 12:2716. doi: 10.3390/diagnostics12112716, PMID: 36359559 PMC9689402

[22] Vaccarella S Franceschi S Bray F Wild CP Plummer M Dal Maso L . Worldwide thyroid-cancer epidemic: the increasing impact of overdiagnosis. N Engl J Med. (2016) 375:614–7. doi: 10.1056/NEJMp1604412, PMID: 27532827

[23] Kaliszewski K Zubkiewicz-Kucharska A Kiełb P . Comparison of the prevalence of incidental and non-incidental papillary thyroid microcarcinoma during 2008–2016: a single-center experience. World J Surg Oncol. (2018) 1:202. doi: 10.1186/s12957-018-1501-8, PMID: 30305094 PMC6180613

[24] Chung WY Chang HS Kim EK Park CS . Ultrasonographic mass screening for thyroid carcinoma: a study in women scheduled to undergo a breast examination. Surg Today. (2001) 31:763–7. doi: 10.1007/s005950170044, PMID: 11686552

[25] Park JS Oh KK Kim EK Chang HS Hong SW . Sonographic screening for thyroid cancer in females undergoing breast sonography. AJR Am J Roentgenol. (2006) 186:1025–8. doi: 10.2214/AJR.04.1659, PMID: 16554573

[26] Derwahl M Nicula D . Estrogen and its role in thyroid cancer. Endocr Relat Cancer. (2014) 21:273–83. doi: 10.1530/ERC-14-0053, PMID: 25052473

[27] Lee JS Lee JS Yun HJ Kim SM Chang H Lee YS . Aggressive subtypes of papillary thyroid carcinoma smaller than 1 cm. J Clin Endocrinol Metab. (2023) 108:1370–5. doi: 10.1210/clinem/dgac739, PMID: 36546348 PMC10188299

[28] Zuhur SS Aggul H Avci U Erol S . Do histologically aggressive subtypes of papillary thyroid microcarcinoma have worse clinical outcome than non-aggressive papillary thyroid microcarcinoma subtypes? A multicenter cohort study. Horm Metab Res. (2023) 55:323–32. doi: 10.1055/a-2032-5810, PMID: 36764327

[29] Wang X Cheng W Liu C Li J . The tall cell variant of papillary thyroid carcinoma: current evidence on clinicopathological features and molecular biology. Oncotarget. (2016) 7:40792–8. doi: 10.18632/oncotarget.8215, PMID: 27008708 PMC5130045

[30] Miyauchi A . Clinical trials of active surveillance of papillary microcarcinoma of the thyroid. World J Surg. (2016) 40:516–22. doi: 10.1007/s00268-015-3392-y, PMID: 26744340 PMC4746213

[31] Orlando G Scerrino G Corigliano A Vitale I Tutino R Radellini S . Papillary thyroid microcarcinoma: active surveillance versus surgery—considerations of an Italian working group from a systematic review. Front Oncol. (2022) 12:859461. doi: 10.3389/fonc.2022.859461, PMID: 35402255 PMC8984605

[32] Kang IK Park J Bae JS Kim JS Kim K . Lymph node ratio predicts recurrence in patients with papillary thyroid carcinoma with low lymph node yield. Cancers (Basel). (2023) 15:2947. doi: 10.3390/cancers15112947, PMID: 37296909 PMC10252081

[33] So YK Kim MJ Kim S Son YI . Lateral lymph node metastasis in papillary thyroid carcinoma: a systematic review and meta-analysis for prevalence, risk factors, and location. Int J Surg. (2018) 50:94–103. doi: 10.1016/j.ijsu.2017.12.029, PMID: 29329789

